# Development and validation of a multivariable mortality risk prediction model for COPD in primary care

**DOI:** 10.1038/s41533-022-00280-0

**Published:** 2022-05-31

**Authors:** Syed A. Shah, Bright I. Nwaru, Aziz Sheikh, Colin R. Simpson, Daniel Kotz

**Affiliations:** 1 Usher Institute, The University of Edinburgh, Edinburgh, UK; 2 Krefting Research Centre, Institute of Medicine, University of Gothenburg, Gothenburg, Sweden; 3 Wallenberg Centre for Molecular and Translational Medicine, Institute of Medicine, University of Gothenburg, Gothenburg, Sweden; 4 School of Health, Wellington Faculty of Health, Victoria University of Wellington, Wellington, New Zealand; 5 Institute of General Practice, Addiction Research and Clinical Epidemiology Unit, Centre for Health and Society (CHS), Medical Faculty of the Heinrich-Heine-University Düsseldorf, Düsseldorf, Germany

**Keywords:** Chronic obstructive pulmonary disease, Epidemiology

## Abstract

Risk stratification of chronic obstructive pulmonary disease (COPD) patients is important to enable targeted management. Existing disease severity classification systems, such as GOLD staging, do not take co-morbidities into account despite their high prevalence in COPD patients. We sought to develop and validate a prognostic model to predict 10-year mortality in patients with diagnosed COPD. We constructed a longitudinal cohort of 37,485 COPD patients (149,196 person-years) from a UK-wide primary care database. The risk factors included in the model pertained to demographic and behavioural characteristics, co-morbidities, and COPD severity. The outcome of interest was all-cause mortality. We fitted an extended Cox-regression model to estimate hazard ratios (HR) with 95% confidence intervals (CI), used machine learning-based data modelling approaches including k-fold cross-validation to validate the prognostic model, and assessed model fitting and discrimination. The inter-quartile ranges of the three metrics on the validation set suggested good performance: 0.90–1.06 for model fit, 0.80–0.83 for Harrel’s c-index, and 0.40–0.46 for Royston and Saurebrei’s $R_D^2$ with a strong overlap of these metrics on the training dataset. According to the validated prognostic model, the two most important risk factors of mortality were heart failure (HR 1.92; 95% CI 1.87–1.96) and current smoking (HR 1.68; 95% CI 1.66–1.71). We have developed and validated a national, population-based prognostic model to predict 10-year mortality of patients diagnosed with COPD. This model could be used to detect high-risk patients and modify risk factors such as optimising heart failure management and offering effective smoking cessation interventions.

## Introduction

An estimated 251 million patients were reported to have chronic obstructive pulmonary disease (COPD) worldwide in 2016^1^, and COPD is expected to become the third leading cause of death by 2030^2^. While efforts to find optimal management of COPD patients continue^3,4^, developing effective management plans remains challenging^5,6^. This is partly because COPD is a heterogeneous and progressive disease with differing pathophysiology. Effective COPD management, thus, requires tools that offer accurate estimates of prognosis over different time horizons that can enhance risk stratification of patients to enable targeted (and more effective) management.

Since spirometry is the most objective tool for measuring airway obstruction and considered essential for COPD diagnosis, the first Global Initiative for Chronic Obstructive Lung Disease (GOLD) COPD classification criteria in 2007^3^ were based on forced expiratory volume in 1 s (FEV1) thresholds only. Over time, these have been revised to include respiratory symptoms and risk of exacerbations^4^. Despite these revisions, the accuracy of prognostic models predicting mortality based on GOLD staging criteria has not improved^6^ and generally remains poor^7^. In addition, COPD patients have a high prevalence of multiple coexisting morbidities^8^, which have so far not been considered in COPD GOLD staging guidelines. Consequently, a few studies have developed separate prognostic models to predict mortality in COPD patients (see Supplementary information). However, with the exception of two recent studies^9,10^, these existing studies are based on limited numbers of patients, have short follow-up periods and are typically from a single centre.

There is thus a need to undertake more robust, multi-centre, large and long-term longitudinal studies that consider existing known or suspected risk factors and symptoms (such as smoking, age, dyspnoea) and individual co-morbidities for the development of a clinically useful risk stratification prognostic model. In the current study, we developed and validated a machine learning-based prediction model of 10-year mortality to help risk-stratify patients, using a national-level, primary care database that addressed several of the aforementioned limitations.

## Methods

### Study design and population

The Clinical Practice Research Database (CPRD) database is a live, longitudinal and growing database of de-identified patient data from primary care practices across the UK. At the time of data request for this study (May 2011), there were 4,780,887 patients from 289 practices that had linked data and acceptable data quality. Of these, 37,485 COPD patients fulfilled the eligibility criteria and were included for subsequent analysis. We designed an open cohort study (no start or end date was specified) where the source population was restricted to only a subset of patients with linked data (mortality and socioeconomic status provided by Office for National Statistics (ONS), UK) and to those patients for whom data were considered of acceptable quality. The source population was further restricted to those with at least five years of follow-up data before the COPD diagnosis date and to have a recorded diagnosis of COPD in those aged ≥35 years.

### Ethics approvals and permissions

CPRD has ethical approval from a multi-centre research ethics committee for all purely observational research using the CPRD database. The protocol of the current study was approved by the Independent Scientific Advisory Committee of CPRD (protocol number 10_084R).

### Ascertainment and definition of predictors

All predictors were identified using codes from the Read clinical classification system, a comprehensive system of clinical concepts classification system that has been used nationally across the UK for almost three decades^11^. The predictors identified in this study were: demographic and behavioural characteristics, co-morbidities, and COPD severity.

Demographic characteristics extracted for each patient were: sex (male or female), age, socioeconomic status (quintile as defined by Index of Multiple Deprivation (IMD)^12^), and occupational exposure (a binary variable that suggested the absence or presence of regular exposure to vapours, dust, gases and fumes due to one’s occupation).

Behavioural characteristics extracted were exercise (whether actively doing any exercises), physical activity limitation (whether severely restricted and finding it difficult to undertake daily activities) and smoking status (never smoker, current or former smoker). Due to the known complexity of deriving smoking status from primary care health records (with occasional counter-intuitive occurrences being observed in the database such as being recorded as a former smoker with previous record of being non-smoker only), we devised a finite state machine (FSM)-based approach that can help derive a patient’s smoking status at the time of COPD diagnosis. FSM-based modelling has been extensively used in logic design^13^ and longitudinal modelling of COPD patients^14^. In an FSM-based modelling approach, a machine is in one of a finite number of states and it then transitions to a new state depending on both the input and the state of the machine (Supplementary Fig. S1 illustrates our FSM approach that can help determine a patient’s smoking status at any time). Briefly, a patient is considered to be a non-smoker, and then the patient is followed over time (from the beginning of the patient’s record) and the patient’s smoking status is updated every time there is a new smoking-related Read code in the patient’s record. This update of the smoking status depends on both the smoking-related code (a smoking-related code can either suggest that the patient is a smoker, former smoker or a non-smoker) and the patient’s previously existing smoking status. The various Read codes to determine a patient’s smoking status are provided in Supplementary information in line with a previous study^15^.

The following co-morbidities were extracted: asthma, history of acute respiratory infections (ARI), pulmonary tuberculosis (TB), anxiety, depression, family history of respiratory diseases, hypertension, heart failure and ischaemic heart disease (IHD).

We derived COPD severity by assessing each patient’s record in the immediate 12 months preceding COPD diagnosis. One of the most widely used COPD severity classifications is the one proposed by GOLD^4^ and is based on spirometry, breathlessness, and history of exacerbations. Spirometry data were recorded in less than 1% of patients in the study cohort. Consequently, we relied on the remaining components that are typically used to assess COPD severity: absence or presence of breathlessness, whether the patient was hospitalised due to a COPD-related cause, and history of COPD exacerbations. We used two different measures for breathlessness: absence or presence of any breathlessness-related code in the last 12 months, and the modified MRC (mMRC) dyspnoea scale (see Supplementary information for codes). For exacerbation history, we used a validated algorithm^16^ to derive the number of exacerbations in the immediate 12 months preceding and including COPD diagnosis date. Briefly, a patient was considered to have had an exacerbation if they had one of the following three events recorded in their primary care record: lower respiratory tract infection or acute exacerbation of COPD; antibiotics and oral corticosteroids (OCS) prescribed together; two or more COPD-related symptoms together with prescription of antibiotics and/or OCS on the same day.

**Fig. 1:**
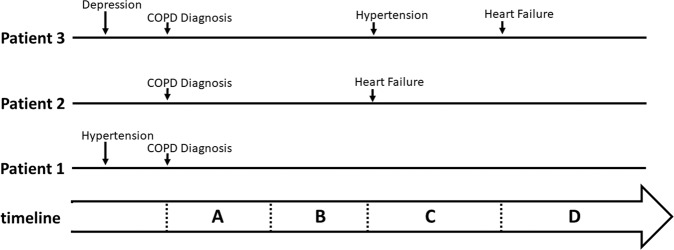
Illustration to show how time-varying exposures are defined. Timeline of three illustrative patients to show how time-varying exposures are defined. Patient 1 is considered to have hypertension at the time of COPD diagnosis and is thus considered to have hypertension in periods A–D. Patient 2 only has COPD in periods A and B but has both COPD and Heart Failure in periods C and D. Patient 3 has COPD and depression in periods A–D, COPD, depression and hypertension in periods C and D, and COPD, depression, hypertension and heart failure in period D.

The variables sex and socioeconomic status were time-invariant (i.e., a single value for each patient that did not change over time). Smoking and COPD severity-associated measures were assessed at baseline, with all remaining variables changing up to once from the time of their first recording. Figure 1 illustrates this concept for three hypothetical patients who had various co-morbidities recorded at different times, relative to the index date (COPD diagnosis date).

### Outcome

The outcome of interest in the study was all-cause mortality (i.e., regardless of whether from COPD or any other reason). Since one of the eligibility criteria was the presence of linked ONS data, the date of mortality were ascertained using ONS source data.

### Statistical analyses

Our eligibility criteria meant that only included complete cases were included (i.e., no missing data). For every patient, an index date was defined, which was the first recorded diagnosis of COPD (as recorded in the general practice electronic health record (EHR)). Every case was labelled as either censored or uncensored. Censored cases were those where a patient was either lost during follow-up or survived at the end of the follow-up period. Uncensored cases were those where a patient had the event of interest (death) during follow-up. Once the censoring information and the time of event (or censoring) were determined for every case, we converted the dataset into a counting process format ensuring that the various exposures in the study (such as ARI, heart failure, etc.) could be time-varying.

**Fig. 2:**
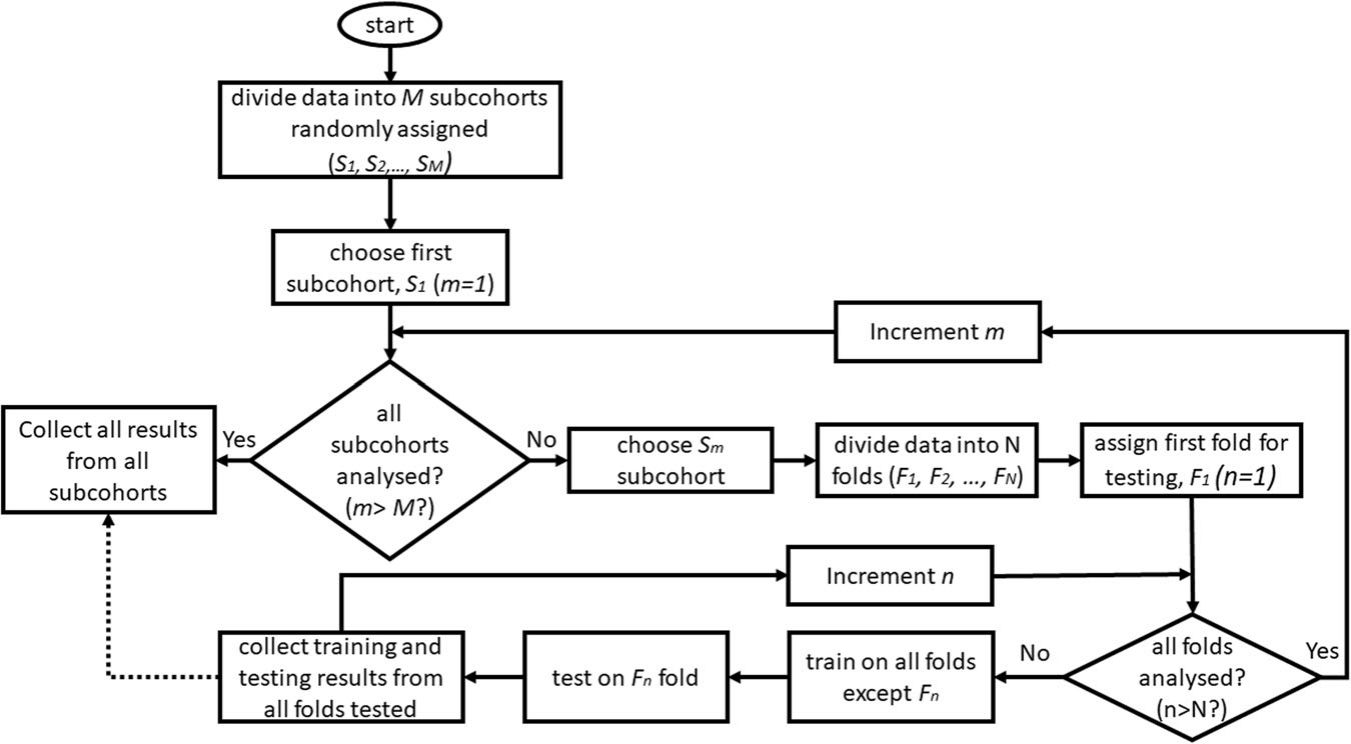
Overview of study methods. Overall flow diagram illustrating how subcohorts were created and how k-fold cross-validation was used for internal validation. In this study, M was chosen to be 8, and N was chosen as 10.

We divided the preprocessed data into subcohorts (randomly sampled from all data without replacement) followed by k-fold cross-validation (Fig. 2)^17^. K-fold cross-validation is one of the most commonly used techniques in machine learning that divide a dataset into *k* groups, develop a model on (*k*−1) groups (the training data), and then validate the model on the remaining *k*th group (the validation test, also known as the testing set)^17^. We developed this methodology to allow us to process the large dataset with internal validation. We applied the extended Cox-regression model using the training data and then evaluated various performance measures (using the trained model) on the validation data.

Performance metrics for survival analysis models can be divided into model fit, discrimination, and calibration^18^. A poorly calibrated model can easily be re-calibrated, and assessing calibration-related performance requires detailed information about the training data (e.g., baseline survival function) that is typically not reported^19^. However, a model with poor discriminative ability cannot be corrected. Consequently, in this work, we used performance metrics associated with model fitting and discrimination for internal validation. For assessing model fit, we estimated the regression coefficient on the risk score (also called the prognostic index). The risk scores were derived from the testing data using the extended Cox-regression coefficients learnt with the training data. Two further metrics to assess model discrimination are the Harrel’s c-index of concordance^20^, and Royston and Saurebrei’s $R_D^2$ measure^21^. The c-index computes the proportion of all patient pairs that are concordant (i.e., a patient with longer survival time is assigned a lower risk score). The $R_D^2$ measures the amount of explained variation on the log relative hazard scale based on the D-statistic proposed by the authors^21^. All confidence intervals in this study were determined at the 0.95 significance level using bootstrapping^22^.

All analyses were performed using the R Studio (version 1.2.5033) and R (version 3.6.2) and the tidyverse packages^23^.

### Study reporting

This manuscript is reported following the recommendation of Transparent Reporting of a multivariable prediction model for Individual Prognosis or Diagnosis (TRIPOD)^24^ and REporting of studies Conducted using Observational Routinely-collected Data (RECORD)^25^.

## Results

### Baseline characteristics of the study population

**Fig. 3:**
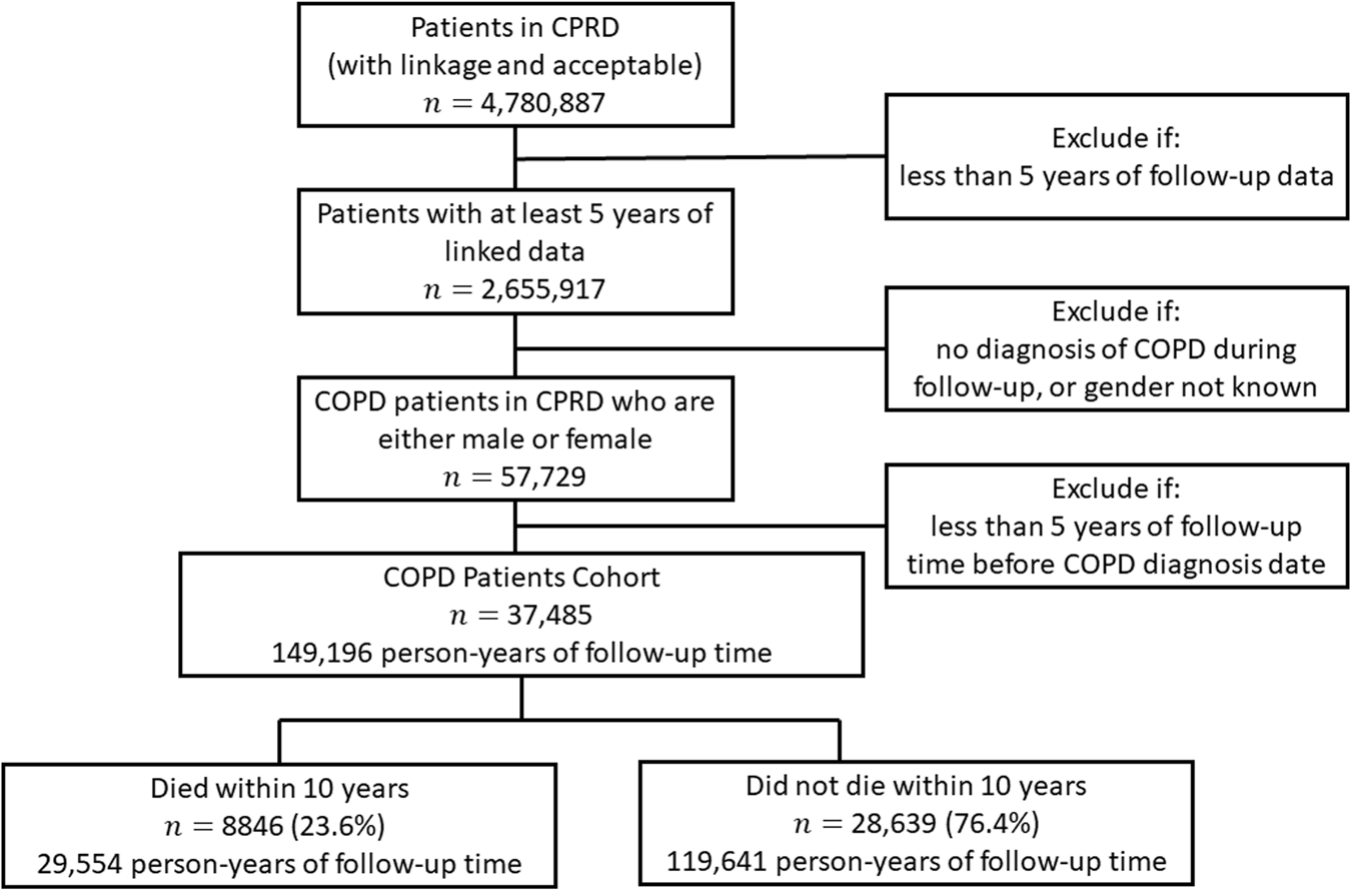
Patient flow diagram. Patient flow diagram with the number of patients with and without the outcome, and a summary of the follow-up time.

Figure 3 describes the flow of participants in the study, a summary of the follow-up time and the number of patients with and without the outcome. The mean age of COPD diagnosis in the study cohort of 37,485 patients was 68 years (see Supplementary information).

The baseline characteristics of these patients are summarised in Table 1. Approximately, a quarter (8846; 23.6%) of patients died within 10 years of COPD diagnosis. Compared to females (3729; 42.2%), a greater proportion of males (5117; 57.8%) died. There was a substantially lower proportion of people in the first quintile of the IMD—i.e., least deprived people—who died compared to those in quintiles 2–5. Furthermore, there was a strong rank correlation between IMD and proportion of patients with COPD who were current smokers: 11.0% in IMD 1 (least deprived), 17.7% in IMD 2, 18.1% in IMD 3, 24.9% in IMD 4 and 28.0% in IMD 5 (most deprived). For the number of exacerbations in a 12-month assessment period prior to and up to including the date of COPD diagnosis, a significantly large proportion of patients (64.3%) did not experience any exacerbation. The mMRC dyspnoea score was also not recorded for almost two-fifths of the patients (38.3%) in the 12 months prior to and up to, including, the COPD diagnosis date. However, for the 61.7% of the patients where mMRC dyspnoea score was recorded at baseline, there was a strong rank correlation between the mMRC dyspnoea score, and the proportion of patients who died: 6.3% when the mMRC dyspnoea score was 0 or 1, 8.5% when the score was 2, and 12.5% when the score was 3 or 4.

**Table 1:** Baseline characteristics of the COPD cohort in the study (total follow-up amounted to 149,196 person-years).

	Frequency	Mortality status within 10 years	
	*N* = 37,485	Alive *n* = 28,639 (76.4%)	Dead *n* = 8846 (23.6%)
Sex			
Male	19,904 (53.1%)	14,787 (51.6%)	5117 (57.8%)
Female	17,581 (46.9%)	13,852 (48.4%)	3729 (42.2%)
Age			
35–45	1045 (2.8%)	1003 (3.5%)	42 (0.5%)
46–55	4140 (11.0%)	3842 (13.4%)	298 (3.4%)
56–70	15,875 (42.4%)	13,393 (46.8%)	2482 (28.1%)
70+	16,425 (43.8%)	10,401 (36.3%)	6024 (68.1%)
Smoking			
Never	5495 (14.7%)	3679 (12.8%)	1816 (20.5%)
Former	23,732 (63.3%)	18,868 (65.9%)	4864 (55.0%)
Current	8258 (22.0%)	6092 (21.3%)	2166 (24.5%)
Index of Multiple Deprivation			
1st quintile (least deprived)	5660 (15.1%)	4442 (15.5%)	1218 (13.8%)
2nd quintile	7606 (20.3%)	5823 (20.3%)	1783 (20.2%)
3rd quintile	7248 (19.3%)	5505 (19.2%)	1743 (19.7%)
4th quintile	8526 (22.7%)	6452 (22.5%)	2074 (23.4%)
5th quintile (most deprived)	8362 (22.3%)	6352 (22.2%)	2010 (22.7%)
Number of exacerbations (within 12 months prior to (and including) the index date)			
0	24,093 (64.3%)	18,590 (64.9%)	5503 (62.2%)
1	8235 (22.0%)	6256 (21.8%)	1979 (22.4%)
≥2	5157 (13.8%)	3793 (13.2%)	1364 (15.4%)
mMRC score (within 12 months prior to (and including) the index date)			
Not recorded	14,349 (38.3%)	7923 (27.7%)	6426 (72.6%)
0–1	8591 (22.9%)	8033 (28.0%)	558 (6.3%)
2	7766 (20.7%)	7012 (24.5%)	754 (8.5%)
3–4	8591 (22.9%)	5671 (19.8%)	1108 (12.5%)

### COPD patients and co-morbidities

Ignoring the time of diagnosis, we found that 30% of patients had no record of any of the co-morbidities considered. For the remaining 70% of patients, asthma, hypertension, and depression were the three most common co-morbidities (see Supplementary Fig. S2 for additional details regarding co-morbidities in the study cohort).

### Risk factors from adjusted extended Cox-regression model

**Fig. 4:**
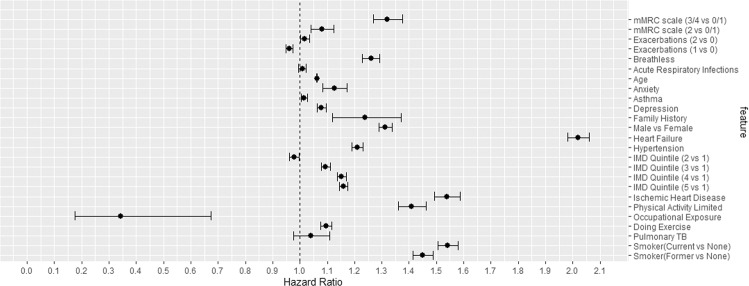
Hazard ratio of various risk factors. Hazard ratio of various risk factors of all-cause mortality (within 10 years) for COPD patients with associated 95% confidence intervals (Supplementary information provides the hazard ratios of all the features, and additional details on how to apply the prognostic model to new patient data).

Figure 4 shows the forest plot of various risk factors of mortality of COPD patients within 10 years (Supplementary information provides additional details). We can see from the figure that the two risk factors of mortality with the largest effect size (also statistically significant) were having heart failure (HR 1.92; 95% CI 1.87–1.96) and being a current smoker (HR 1.68; 95% CI 1.66–1.71). The other risk factors with large effect sizes that were also significant were being diagnosed with IHD (HR 1.49; 95% CI 1.45–1.54), being a former smoker (HR 1.49; 95% CI 1.46–1.51), being male (HR 1.26; 95% CI 1.25–1.27), having an mMRC dyspnoea score of ≥ 3 (HR 1.33; 95% CI 1.28–1.39), breathlessness (HR 1.25; 95% CI 1.24–1.26) and having a physical activity limitation (HR 1.37; 95% CI 1.33–1.42).

### Validation performance

There was greater variability in the distribution of the three performance metrics (model fit, c-index and $R_D^2$) in the validation set compared to the training set, but a strong overlap (Supplementary Fig. S4 provides the distribution of model fit, c-index and $R_D^2$ for both the training and validation set). The inter-quartile ranges of the three performance metrics were: 0.90–1.06 for model fit, 0.80–0.83 for c-index, and 0.40–0.46 for $R_D^2$.

## Discussion

The most significant risk factors associated with mortality (with at least a 50% increased risk) after COPD diagnosis were having heart failure and being a current smoker (compared to non-smoker). Other risk factors (≥20% increased risk of mortality) were: IHD, being a former smoker (compared to non-smoker), being male, restricted physical activity, breathlessness being present as a symptom, and having an mMRC dyspnoea score ≥3 in the 12 months leading up to COPD diagnosis. A small but noteworthy proportion of patients (15%) were diagnosed with COPD despite no history of smoking recorded in primary care. According to the fully adjusted model, a strong rank correlation between socioeconomic status and risk of mortality was observed (i.e., the more deprived a patient, the greater the risk of mortality). Hypertension, anxiety, having a history of pulmonary TB, and depression were also associated with an increased risk of mortality, but their effect was relatively modest (≤20%) in the fully adjusted model. Having a history of ARIs or being diagnosed with asthma was not associated with an increased risk of mortality. The extended Cox-regression model developed in this study was internally validated and achieved strong validation performance.

To our knowledge, this is the largest longitudinal, multi-centre study developing a 10-year mortality risk prediction model with time-varying covariates that includes individual co-morbidities in addition to several suspected risk factors typically available in a primary care setting. While EHRs are a rich resource for clinical research, not every clinical feature is directly coded (e.g., a subset of COPD exacerbations is inferred from a combination of prescription and symptoms data). There are also features (such as smoking) that, although directly coded in EHRs, are prone to error (many examples of patients coded as non-smokers found despite being previously recorded as current/former smoker). We used robust data modelling approaches to derive features from EHRs. This includes the use of a validated algorithm to derive COPD exacerbations from prescription and symptom data, and an FSM model to derive patient smoking status. Furthermore, this study developed and illustrated the use of data processing steps that can apply and internally validate statistical models on large datasets.

One limitation of the study was the lack of access to secondary care data, and therefore some hospitalisation episodes not recorded in primary care may have been missed. While it is not possible to determine the number of such cases, we believe that a proportion of such occurrences would have already been accounted for in the primary care dataset since all hospitalisations are accompanied by a discharge letter that gets sent from hospital to primary care and gets added to the patient’s primary care record via a National Health Service document management system. However, it is not possible to ascertain the extent of hospitalisation episodes that actually gets coded in primary care using a discharge letter. Another limitation of the study pertains to the assessment of baseline severity used as a potential risk factor. It is possible that some patients may have been assigned different codes (unrelated to COPD) in the 12 months preceding the COPD diagnosis date. This might explain why COPD exacerbation was not a significant risk factor and why most of the patients (64.3%) did not experience any exacerbation episode during the 12 months immediately prior to the COPD diagnosis date. Another limitation in the study was the lack of FEV1 data available in the primary care records and the baseline severity was therefore based on symptoms and exacerbation only. Lastly, the model was developed on data from the UK only.

Our literature review identified 20 previous studies (see Supplementary information) that developed a prognostic model for mortality prediction. Most of these studies were based on a limited number of COPD patients (less than 1000) and typically came from a single centre. Of the studies with at least 1000 patients, the study by Marin et al.^26^ undertaken on 3633 patients from Spain investigated the use of several multi-component indices based on different combinations of age, dyspnoea, FEV1, body mass index (BMI), exercise capacity and exacerbations to predict 10-year mortality of COPD patients. They found that indices based on age, dyspnoea, FEV1 and BMI were the most useful, and they did not find the additional value of exacerbations when incorporated into the multi-component score. A study by Keene et al.^27^ on 1892 patients found that the multi-component score based on age, dyspnoea and FEV1 provided promising discrimination for predicting 3-year mortality. These studies, however, did not take individual co-morbidities into account, a limitation as corroborated by Aramburu et al.^28^ who found that the prognostic capacity of the existing risk scores improves when co-morbidity information is added but recommended that there is a need for further studies to clarify which co-morbidities need to be taken into account. Consequently, two recent large-scale studies^9,10^, have attempted to develop a mortality prediction model that use routinely available data in primary care in the UK including various individual co-morbidities. However, neither of these studies accounted for any co-morbidity diagnosed after COPD diagnosis, and the model is therefore only relevant to risk-stratify patients at the time (or near the time) of COPD diagnosis. In contrast, our study incorporates co-morbidity and its timing (even if it occurs long after a COPD diagnosis) in the model and it is therefore applicable to assess the risk of a COPD patient any time during follow-up. In addition, our study predicts mortality over a longer time-horizon—i.e., 10 years—as opposed to the two previous studies (1 year in Ref. ^9^ and 5 years in Ref. ^10^).

We also found that approximately 14% patients diagnosed with COPD were non-smokers. This finding agrees with a previous study^29^ that found a substantial proportion of COPD patients (22% in their case) who had no history of smoking. That study also found that COPD patients with no history of smoking tend to have a milder disease, in line with our finding that both former and current smoking are significant risk factors for mortality, compared to being a non-smoker.

This study clearly demonstrates that various co-morbidities increase the risk of mortality in patients diagnosed with COPD and underlines the need for a comprehensive COPD management strategy that takes co-morbidities into account. Not all co-morbidities modify mortality risk of COPD patients equally. Patients with specific co-morbidities (such as heart failure and IHD) should be prioritised for targeted interventions to help improve their chances of survival. Even when co-morbidities are accounted for, smoking remains an important risk factor for mortality in COPD patients and therefore, patients should be offered effective smoking cessation interventions. While the model developed in the study was validated internally, there is now a need to validate this in a comparable, external dataset and prospectively evaluate its performance in routine care. External validation will provide an opportunity to test the model’s performance on an independent patient population and thus provide a stronger evidence base to help negotiate regulatory requirements for deployment^30^.

In summary, we have developed and validated a prognostic model to predict the 10-year mortality of patients diagnosed with COPD using national-level, primary care data (the largest study on the topic to date). The prognostic model found several risk factors to be associated with 10-year mortality, with varying degrees of strength. The most significant of these factors were heart failure and being a current smoker; in contrast, history of ARIs and asthma were not independent risk factors of poor prognosis. While the model developed achieved strong internal validation performance, it is now important to externally validate this model before deploying it in clinical practice.

### Reporting summary

Further information on research design is available in the Nature Research Reporting Summary linked to this article.

## Supplementary information

Supplementary Material

Reporting Summary

## Data Availability

The data used in this study are not publicly available. However, the data can be requested from CPRD (a third-party entity; www.cprd.com) subject to an independent evaluation of scientific protocol.

## Code availability

The code used in this study will be made available at https://github.com/syedahmar/COPD-Mortality-Prediction under Creative Commons License.
