# Publisher Correction: Development and validation of a multivariable mortality risk prediction model for COPD in primary care

**DOI:** 10.1038/s41533-022-00284-w

**Published:** 2022-06-15

**Authors:** Syed A. Shah, Bright I. Nwaru, Aziz Sheikh, Colin R. Simpson, Daniel Kotz

**Affiliations:** 1 Usher Institute, The University of Edinburgh, Edinburgh, UK; 2 Krefting Research Centre, Institute of Medicine, University of Gothenburg, Gothenburg, Sweden; 3 Wallenberg Centre for Molecular and Translational Medicine, Institute of Medicine, University of Gothenburg, Gothenburg, Sweden; 4 School of Health, Wellington Faculty of Health, Victoria University of Wellington, Wellington, New Zealand; 5 Institute of General Practice, Addiction Research and Clinical Epidemiology Unit, Centre for Health and Society (CHS), Medical Faculty of the Heinrich-Heine-University Düsseldorf, Düsseldorf, Germany

**Keywords:** Chronic obstructive pulmonary disease, Epidemiology

Correction to: *npj Primary Care Respiratory Medicine* 10.1038/s41533-022-00280-0, published online 31 May 2022

In this article the caption to Figs. 1–4 was inadvertently swapped. The original article has been corrected.

