# Supplementary material for: Development and validation of a multivariable mortality risk prediction model for COPD in primary care
Source: NPJ Prim Care Respir Med. 2022 May 31;32:21. doi: 10.1038/s41533-022-00280-0 (PMC9156666; doi:10.1038/s41533-022-00280-0)
Supplement: Supplementary file 1 — Supplementary Material [file 41533_2022_280_MOESM1_ESM.pdf]

Development and Validation of a Multivariable Mortality Risk Prediction Model for COPD in Primary Care

Supplementary Notes

1. Previous models on mortality prediction for COPD

We searched PubMed for research studies for all studies with titles that contained the relevant terms for population ("COPD") together with outcome ("death" OR "mortality" OR "survival") and method ("machine learning" OR "model" OR "artificial intelligence" OR "predict" OR "predictors" OR "prediction"). Our search terms yielded 55 articles published in the last 10 years. Of these studies, some studies only focussed on specific COPD patients (such as those requiring hospitalization or having severe COPD). There were, however, 20 articles that reported the development of a prognostic model for mortality in COPD patients.

For these 20 articles, Table 1 summarises the number of patients, and the time-horizon over which the mortality prediction was based.

	Number of Patients	Time-Horizon
(Prudente *et al.*, 2018)	133	9 years
(Çiftçi *et al.*, 2019)	42	8 years
(Brusse-Keizer *et al.*, 2017)	795	3 years
(Loh *et al.*, 2018)	112	2.7 years (median follow-up time)
(Maters *et al.*, 2014)	224	4.2 years (median follow-up time)
(Morales *et al.*, 2018)[1]	54,789	1-3 years
(Vieira *et al.*, 2020)	186	4.1 years (mean follow-up time)
(Almagro *et al.*, 2014)	3,633	5 years
(Marin *et al.*, 2013)	3,633	10 years
(Henoch *et al.*, 2020)[2]	39,830	Not reported
(Nyssen *et al.*, 2013)	30	Not reported in abstract (article not in English)
(Ryynänen *et al.*, 2013)	738	1-3 years
(Aramburu *et al.*, 2019)	543	5 years
(Cardoso *et al.*, 2018)	98	5 years
(Liu *et al.*, 2011)	114	Up to 3.3 years
(Ozgür *et al.*, 2012)	73	3.9 years (median follow-up time)
(Stolz *et al.*, 2014)	385	3 and 5 years
(Brat *et al.*, 2020)	699	Not available
(Kiddle *et al.*, 2020)	48,304	5 years
(Bloom *et al.*, 2019)	54,990	1 year

[1]This study did not develop any prediction model, and only attempted to validate existing models

[2]This study, although large, did not use COPD diagnosis time as index date and follow-up period after diagnosis date was therefore unclear.

References

Almagro, P. *et al.* (2014) 'Finding the best thresholds of FEV1 and dyspnea to predict 5-year survival in COPD patients: the COCOMICS study.', *PloS one*, 9(2), p. e89866. doi: 10.1371/journal.pone.0089866.

Aramburu, A. *et al.* (2019) 'COPD classification models and mortality prediction capacity', *International journal of chronic obstructive pulmonary disease*. Dove Press, 14, p. 605.

Bloom, C. I. *et al.* (2019) 'Predicting COPD 1-year mortality using prognostic predictors routinely measured in primary care.', *BMC medicine*, 17(1), p. 73. doi: 10.1186/s12916-019-1310-0.

Brat, K. *et al.* (2020) 'Introducing a new prognostic instrument for long-term mortality prediction in COPD patients: the CADOT index.', *Biomedical papers of the Medical Faculty of the University Palacky, Olomouc, Czechoslovakia*. Czech Republic. doi: 10.5507/bp.2020.035.

Brusse-Keizer, M. *et al.* (2017) 'Comparing the 2007 and 2011 GOLD Classifications as Predictors of all-Cause Mortality and Morbidity in COPD.', *COPD*. England, 14(1), pp. 7–14. doi: 10.1080/15412555.2016.1206875.

Cardoso, J. *et al.* (2018) 'Prediction of severe exacerbations and mortality in COPD: the role of exacerbation history and inspiratory capacity/total lung capacity ratio.', *International journal of chronic obstructive pulmonary disease*, 13, pp. 1105–1113. doi: 10.2147/COPD.S155848.

Çiftçi, F. *et al.* (2019) 'Does exercise capacity, dyspnea level, or quality of life actually predict mortality in patients with COPD? 8-year follow-up.', *Tuberkuloz ve toraks*. Turkey, 67(2), pp. 83–91. doi: 10.5578/tt.67725.

Henoch, I. *et al.* (2020) 'Early Predictors of Mortality in Patients with COPD, in Relation to Respiratory and Non-Respiratory Causes of Death - A National Register Study.', *International journal of chronic obstructive pulmonary disease*, 15, pp. 1495–1505. doi: 10.2147/COPD.S252709.

Kiddle, S. J. *et al.* (2020) 'Prediction of five-year mortality after COPD diagnosis using primary care records.', *PloS one*, 15(7), p. e0236011. doi: 10.1371/journal.pone.0236011.

Liu, S.-F. *et al.* (2011) 'High value of combined serum C-reactive protein and BODE score for mortality prediction in patients with stable COPD.', *Archivos de bronconeumologia*. Spain, 47(9), pp. 427–432. doi: 10.1016/j.arbres.2011.04.011.

Loh, L.-C. *et al.* (2018) 'A novel CT-emphysema index/FEV(1) approach of phenotyping COPD to predict mortality.', *International journal of chronic obstructive pulmonary disease*, 13, pp. 2543–2550. doi: 10.2147/COPD.S165898.

Marin, J. M. *et al.* (2013) 'Multicomponent indices to predict survival in COPD: the COCOMICS study', *European Respiratory Journal*. Eur Respiratory Soc, 42(2), pp. 323–332.

Maters, G. A. *et al.* (2014) 'Predictors of all-cause mortality in patients with stable COPD: medical co-morbid conditions or high depressive symptoms.', *COPD*. England, 11(4), pp. 468–474. doi: 10.3109/15412555.2014.898026.

Morales, D. R. *et al.* (2018) 'External validation of ADO, DOSE, COTE and CODEX at predicting death in primary care patients with COPD using standard and machine learning approaches.', *Respiratory medicine*. England, 138, pp. 150–155. doi: 10.1016/j.rmed.2018.04.003.

Nyssen, S. M. *et al.* (2013) 'Levels of physical activity and predictors of mortality in COPD.', *Jornal brasileiro de pneumologia : publicacao oficial da Sociedade Brasileira de Pneumologia e Tisilogia*, 39(6), pp. 659–666. doi: 10.1590/S1806-37132013000600004.

Ozgür, E. S. *et al.* (2012) 'An integrated index combined by dynamic hyperinflation and exercise capacity in the prediction of morbidity and mortality in COPD.', *Respiratory care*. United States, 57(9), pp. 1452–1459. doi: 10.4187/respcare.01440.

Prudente, R. *et al.* (2018) 'Predictors of mortality in patients with COPD after 9 years.', *International journal of chronic obstructive pulmonary disease*, 13, pp. 3389–3398. doi: 10.2147/COPD.S174665.

Ryynänen, O.-P. *et al.* (2013) 'Bayesian predictors of very poor health related quality of life and mortality in patients with COPD.', *BMC medical informatics and decision making*, 13, p. 34. doi: 10.1186/1472-6947-13-34.

Stolz, D. *et al.* (2014) 'Mortality risk prediction in COPD by a prognostic biomarker panel.', *The European respiratory journal*. England, 44(6), pp. 1557–1570. doi: 10.1183/09031936.00043814.

Vieira, E. B. *et al.* (2020) 'Modified BODE Index to Predict Mortality in Individuals With COPD: The Role of 4-Min Step Test.', *Respiratory care*. United States, 65(7), pp. 977–983. doi: 10.4187/respcare.06991.

2. Illustration of the Finite-State Machine (FSM) model to derive smoking status from longitudinal primary care record

In a Finite State Machine-based modelling approach, a machine is in one of a finite number of states and it then transitions to a new state depending on both the input and the state of the machine. Briefly, a patient is considered to be a non-smoker, and then the patient is followed over time (from the beginning of the patient's record) and the patient's smoking status is updated every time there is a new smoking-related Read code in the patient's record. This update of the smoking status depends on both the smoking-related code (a smoking-related code can either suggest that the patient is a smoker, former smoker or a non-smoker) and the patient's previously existing smoking status.

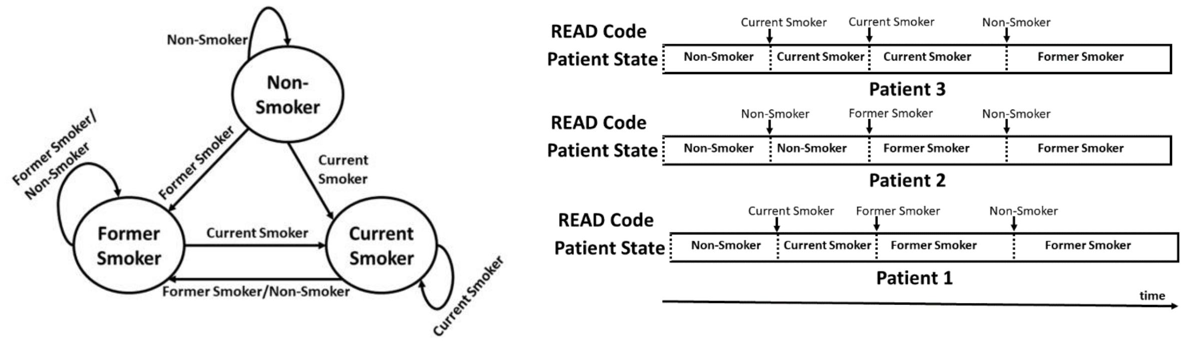

(a): FSM model for determining a patient's smoking status

(b): Illustration of applying FSM model to 3 hypothetical patients

Supplementary Figure 1: Finite State Machine-based smoking status determination and corresponding illustration(a) A patient can be in any of the three states (illustrated by the circles) at any given time: non-smoker, current smoker, or former smoker. A patient's record can, at any time, include a code associated with a "former smoker", "current smoker" or "non-smoker" illustrated by the arrows. Depending on a patient's current state, any new patient record will determine the patient's new state. (b) All patients are considered as non-smokers unless there is a smoking-related code. Patient 1 first switches to current smoker from non-smoker (after a current smoker code), then switches to former smoker after having a former smoker code and a non-smoker code at a subsequent date. Patient 2 stays as non-smoker after having a non-smoker code. Subsequently, however, patient 2 switches to a former smoker status after having a former smoker code

and stays as former smoker despite getting a non-smoker code later. Applying the same FSM model, patient 3 switches to a current smoker from non-smoker and stays as current smoker until a new code of non-smoker switches this patient to a former smoker.

Read Codes for Non-Smoker

1371.	Never smoked tobacco
9kn..	Non-smoker annual review - enhanced services administration

Read Codes for Former Smoker

137K.	Stopped smoking
137L.	Current non-smoker
137N.	Ex pipe smoker
137O.	Ex cigar smoker
137S.	Ex smoker
137T.	Date ceased smoking
1377.	Ex-trivial smoker (< 1 per day)
1378.	Ex-light smoker (1 - 9 per day)
1379.	Ex-moderate smoker (10 - 19 per day)
137A.	Ex-heavy smoker (20 - 39 per day)
137B.	Ex-very heavy smoker (40 + per day)
137F.	Ex-smoker - amount unknown
137i.	Ex tobacco chewer
137j.	Ex-cigarette smoker
137K0	Recently stopped smoking
9km..	Ex-smoker annual review - enhanced services administration
13p4.	Smoking free weeks
137l.	Ex roll-up cigarette smoker

Read Codes for Current Smoker

(%: wildcard entries)

745H%	(Various) Smoking cessation therapy
du3%	(Various) Nicotine replacement therapy
du6%	(Various) Bupropion
du7%	(Various) additional nicotine replacement therapy
du8%	(Various) Varenicline
du9%	(Various) Nicotine withdrawal products
E251%	(Various) tobacco dependence
137..	Tobacco consumption
137Z	Tobacco consumption NOS
137X.	Cigarette consumption
137Y.	Cigar consumption
137E.	Tobacco consumption unknown
137g.	Cigarette pack years
1372.	Trivial smoker - < 1 per day
1373.	Light smoker - 1-9 per day
1374.	Moderate smoker - 10-19 per day
1375.	Heavy smoker - 20-39 per day
1376.	Very heavy smoker - 20-39 per day
137a.	Pipe tobacco consumption
137b.	Ready to stop smoking
137C.	Keeps trying to stop smoking
137c.	Thinking about stopping smoking

137e.	Smoking restarted
137G.	Trying to give up smoking
137H.	Pipe smoker
137J.	Cigar smoker
137M.	Rolls own cigarettes
137P.	Cigarette smoker
137Q.	Smoking started
137R.	Current smoker
137V.	Smoking reduced
137D.	Admitted tobacco cons untrue ?
137d.	Not interested in stopping smoking
137f.	Reason for restarting smoking
137h.	Minutes from waking to first tobacco consumption
6791.	Health ed. - smoking
67910	Health education - parental smoking
137m.	Failed attempt to stop smoking
13p..	Smoking cessation milestones
13p0.	Negotiated date for cessation of smoking
13p8.	Lost to smoking cessation follow-up
38DH.	Fagerstrom test for nicotine dependence
67A3.	Pregnancy smoking advice
67H1.	Lifestyle advice regarding smoking
67H6.	Brief cessation for smoking cessation
8B2B.	Nicotine replacement therapy

8B3f.	Nicotine replacement therapy provided free
8B3Y.	Over the counter nicotine replacement therapy
8BP3.	Nicotine replacement therapy provided by community pharmacis
8CAg.	Smoking cessation advice provided by community pharmacist
8CAL.	Smoking cessation advice
8CdB.	Stop smoking service opportunity signposted
8H7i.	Referral to smoking cessation advisor
8HBM.	Stop smoking face to face follow-up
8HkQ.	Referral to NHS stop smoking service
8HTK.	Referral to stop-smoking clinic
8I2I.	Nicotine replacement therapy contraindicated
8I2J.	Bupropion contraindicated
8I39.	Nicotine replacement therapy refused
8I3M.	Bupropion refused
8I6H.	Smoking review not indicated
8IAj.	Smoking cessation advice declined
8IEK.	Smoking cessation program declined
8IEM.	Smoking cessation drug therapy declined
9hG..	Exception reporting: smoking quality indicators
9hG0.	Excepted from smoking quality indicators: Patient unsuitable
9hG1.	Excepted from smoking quality indicators: Informed dissent
9kc..	Smoking cessation - enhanced services administration
9kc0.	Smoking cessatn monitor template complet - enhanc serv admin
9ko..	Current smoker annual review - enhanced service admin

9N2k.	Seen by smoking cessation advisor
9N4M.	DNA - did not attend smoking cessation clinic
9Ndg.	Declined consent for follow-up by smoking cessation team
9NdV.	Consent given follow-up after smoking cessation intervention
9NdW.	Consent given for smoking cessation data sharing
9NdY.	Declin cons follow-up evaluation after smoking cess interven
9NdZ.	Declined consent for smoking cessation data sharing
9NS02	Referral for smoking cessation service offered
9OO..	Attends stop smoking monitor admin
9OO1.	Attends stop smoking monitor
9OO2.	Refuses stop smoking monitor
9OO3.	Stop smoking monitor default
9OO4.	Stop smoking monitor 1st lettr
9OO5.	Stop smoking monitor 2nd lettr
9OO6.	Stop smoking monitor 3rd lettr
9OO7.	Stop smoking monitor verb.inv.
9OO8.	Stop smoking monitor phone inv
9OO9.	Stop smoking monitoring delete
9OOA.	Stop smoking monitor check.done
9OOB.	Stop smoking invitation short message service text message
9OOB0	Stop smoking invitation first SMS text message
9OOB1	Stop smoking invitation second SMS text message
9OOB2	Stop smoking invitation third SMS text message
9OOZ.	Stop smoking monitor admin.NOS

E023.	Nicotine withdrawal
J0364	Tobacco deposit on teeth
SMC..	Toxic effect of tobacco and nicotine
TJHy2	Adverse reaction to nicotine
U6099	[X] Bupropion causing adverse effects in therapeutic use
ZV4K0	[V] Tobacco use
ZV6D8	[V] Tobacco abuse counselling
13p5.	Smoking cessation programme start date
9ko..	Current smoker annual review - enhanced service admin

3. Other Read Codes used in the study

Read/Medcodes to derive COPD severity associated measures:

(i) mMRC dyspnoea scale
(ii) Breathlessness
(iii) Hospitalization
(iv) Exacerbation as defined in (Rothnie *et al.*, 2016)

(i) <u>Read codes for mMRC dyspnoea scale</u>

Read Code	Description
XaIUi	mMRC Breathlessness Scale: 0
XaIUl	mMRC Breathlessness Scale: 1
XaIUm	mMRC Breathlessness Scale: 2
XaIUn	mMRC Breathlessness Scale: 3
XaIUo	mMRC Breathlessness Scale: 4

(ii) <u>Medcodes for Breathlessness</u>

Medcode	Description
735	[D]Breathlessness
741	[D]Shortness of breath
1429	Breathlessness
2563	[D]Respiratory distress
2575	Short of breath on exertion
2737	Respiratory distress syndrome
2931	Difficulty breathing

3092	[D]Dyspnoea
4822	Shortness of breath
5175	Breathlessness symptom
5349	Shortness of breath symptom
5896	Dyspnoea - symptom
6326	Breathless - moderate exertion
6434	Paroxysmal nocturnal dyspnoea
7000	O/E - dyspnoea
7534	O/E - respiratory distress
7683	Breathless - lying flat
7932	Breathless - mild exertion
9297	[D]Respiratory insufficiency
18116	Nocturnal dyspnoea
21801	Breathlessness NOS
22094	Short of breath dressing/undressing
24889	Breathless - strenuous exertion
31143	Breathless - at rest
40813	Unable to complete a sentence in one breath
53771	Dyspnoea on exertion

(iii) Medcodes for Hospitalization

medcode	description
46036	multiple copd emergency hospital admissions
19003	emergency copd admission since last appointment
19106	copd accident and emergency attendance since last visit
11019	admit copd emergency

(iv) Medcodes for exacerbation

Refer to supplementary information provided by (Rothnie *et al.*, 2016) that lists the medcodes associated with lower respiratory tract infection (LRTI), acute exacerbation of COPD (AECOPD), antibiotics, oral cortico-steroids (OCS), COPD symptoms (cough, sputum, breathlessness) and annual review.

Reference

Rothnie, K. J. *et al.* (2016) 'Validation of the recording of acute exacerbations of COPD in UK primary care electronic healthcare records', *PLoS one*. Public Library of Science San Francisco, CA USA, 11(3), p. e0151357.

4. Additional Figures

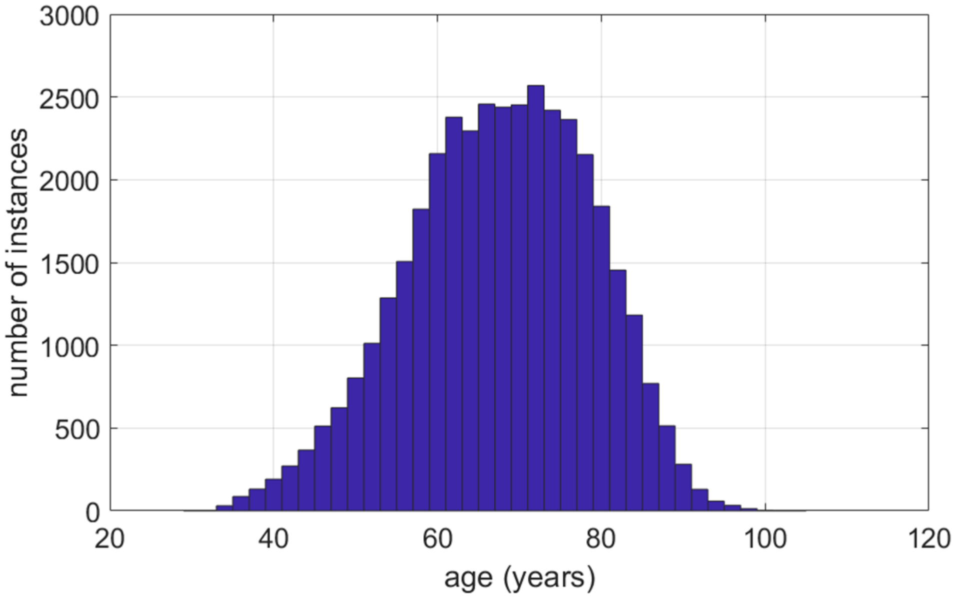

Supplementary Figure 2: Distribution of age at which COPD diagnosis was made (mean age of COPD diagnosis was 68 ± 11)

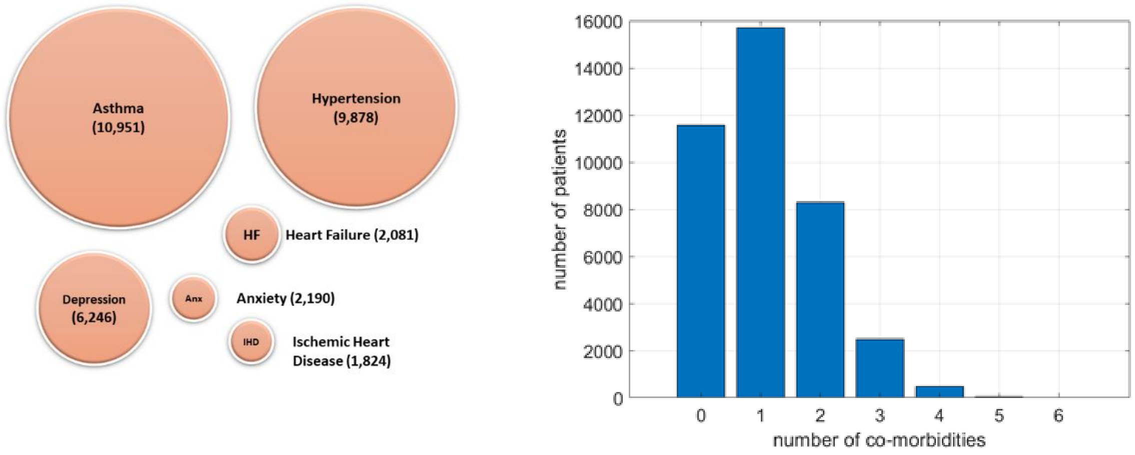

Supplementary Figure 3: The 6 most common co-morbidities in our cohort of COPD patients and the distribution of number of co-morbidities

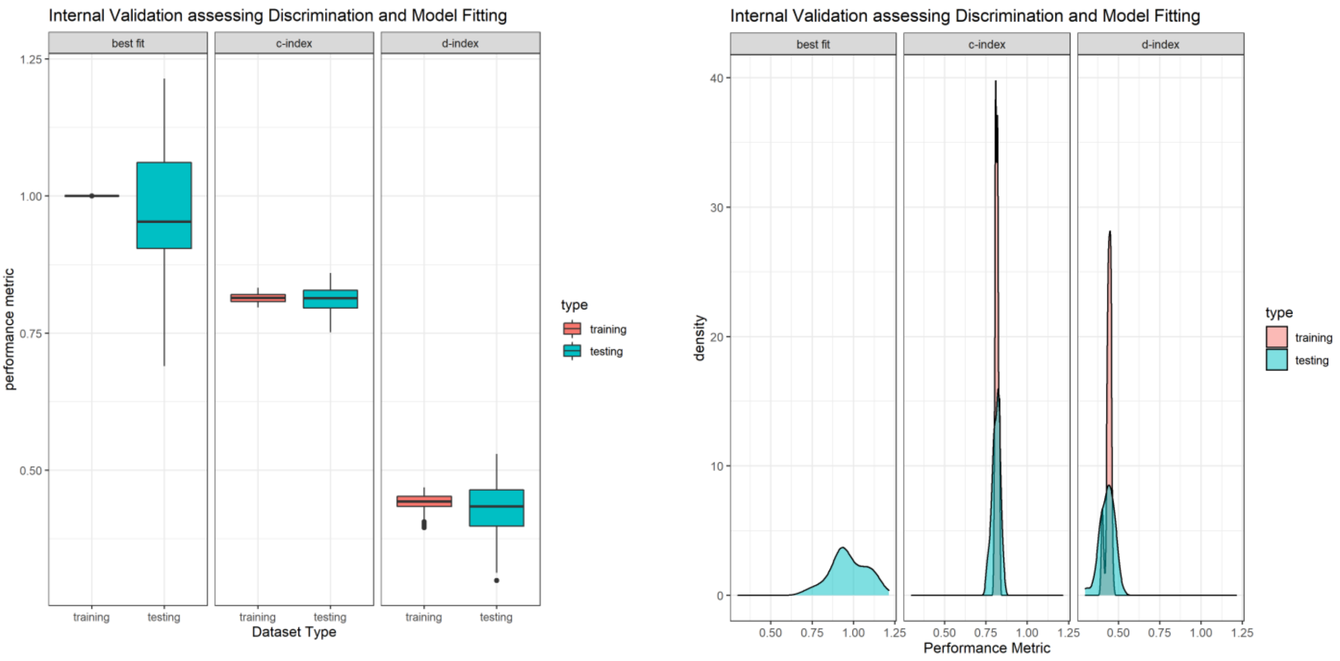

Supplementary Figure 4: Distribution of the various internal validation measures in both the training set and testing set

5. How to use the model?

The extended Cox Regression model consists of coefficients associated with both time-independent and time-dependent features (see equation 1).

$$h(t, \boldsymbol{X}(t)) = h_0(t) \exp\left[\sum_{i=1}^{p_1} B_i X_i + \sum_{j=1}^{p_2} B_i X_j(t)\right] \quad (1)$$

$$PI(t) = \exp\left[\sum_{i=1}^{p_1} B_i X_i + \sum_{j=1}^{p_2} B_i X_j(t)\right] \quad (2)$$

$$h(t, \boldsymbol{X}(t)) = h_0(t) PI(t) \quad (3)$$

In equation (1), $h(t, \boldsymbol{X}(t))$ refers to the value of hazard function at time t, $\boldsymbol{X}(t)$ refers to patient's set of features at time t, $h_0(t)$ represents the baseline hazard function, $B_i X_i$ refers to the p_1 feature components that are time-independent, and $B_i X_j$ refer to the p_2 feature components that are time-dependent. To apply the model developed in our study, it suffices to calculate the prognostic index,$PI(t)$, part of the equation (equation 2) which is independent of the baseline hazard function, $h_0(t)$, and can still allow us to assess the model's discrimination and model-fitting characteristics. To evaluate the mortality risk of a test patient at a specific time (say t) using the extended Cox Regression model, calculate the prognostic risk score, $PI(t)$, of the patient.

Supplementary Table 1 lists the hazard ratios and the associated 95% confidence interval bounds. To calculate $PI(t)$, the hazard ratios need to be converted to coefficients of the

extended Cox regression model. This can be achieved by taking the logarithm of each value provided in the table.

Supplementary Table 1: Exponent of the Regression Coefficients of the extended Cox Regression Model (BMI: kg/m^2)

Feature	Mean	95% CI Lower Bound	95% CI Upper Bound
Smoker(Never)	1.00	1.00	1.00
Smoker(Former)	1.45	1.49	1.42
Smoker(Current)	1.54	1.58	1.51
Pulmonary TB	1.04	1.11	0.98
Exercise	1.10	1.12	1.07
Occupational Exposure	0.34	0.67	0.18
No Physical Activity	1.41	1.46	1.36
Ischemic Heart Disease	1.54	1.59	1.49
IMD 5th Quintile	1.16	1.17	1.15
IMD 4th Quintile	1.15	1.17	1.14
IMD 3rd Quintile	1.09	1.11	1.08
IMD 2nd Quintile	0.98	1.00	0.96
IMD 1st Quintile	1.00	1.00	1.00
Hypertension	1.21	1.23	1.19
Heart Failure	2.02	2.06	1.98
Gender: Male	1.31	1.34	1.29
Gender: Female	1.00	1.00	1.00
Family History	1.24	1.37	1.12
Depression	1.08	1.10	1.06
Asthma	1.02	1.03	1.00
Anxiety	1.13	1.17	1.08
Age	1.06	1.06	1.06
Acute Respiratory Infections	1.01	1.02	1.00
Breathless	1.26	1.29	1.23
Exacerbations (0)	1.00	1.00	1.00
Exacerbations (1)	0.96	0.97	0.95
Exacerbations (2)	1.02	1.03	1.00
mMRC Scale (<2)	1.00	1.00	1.00
mMRC Scale (2)	1.08	1.12	1.04
mMRC Scale (3 or more)	1.32	1.38	1.27
BMI (20-24.99)	1.00	1.00	1.00
BMI (under 20)	1.46	1.52	1.41
BMI (25-30)	0.80	0.82	0.79
BMI (over 30)	0.86	0.87	0.84
